# Enzalutamide response in a panel of prostate cancer cell lines reveals a role for glucocorticoid receptor in enzalutamide resistant disease

**DOI:** 10.1038/s41598-020-78798-x

**Published:** 2020-12-10

**Authors:** Rebecca Smith, Moqing Liu, Tiera Liby, Nora Bayani, Elmar Bucher, Kami Chiotti, Daniel Derrick, Anne Chauchereau, Laura Heiser, Joshi Alumkal, Heidi Feiler, Peter Carroll, James E. Korkola

**Affiliations:** 1 Department of Biomedical Engineering, Oregon Health & Science University, 2730 SW Moody Ave CLSB Rm 3N018, Portland, OR 97201 USA; 2 Life Sciences Division, Lawrence Berkeley National Laboratories, Berkeley, CA 94720 USA; 3 Department of Molecular and Medical Genetics, Oregon Health & Science University, Portland, OR 97201 USA; 4 INSERM U981, Gustave Roussy Institute, Paris-Saclay University, 94800 Villejuif, France; 5 OHSU Center for Spatial Systems Biomedicine, Oregon Health & Science University, Portland, OR 97201 USA; 6 Department of Medicine, Oregon Health & Science University, Portland, OR 97201 USA; 7 Department of Urology, UCSF, San Francisco, CA 94158 USA; 8 Present Address: Department of Internal Medicine, Rogel Cancer Center, University of Michigan, Ann Arbor, MI 48109 USA

**Keywords:** Cancer, Cancer genomics, Urological cancer

## Abstract

Representative in vitro model systems that accurately model response to therapy and allow the identification of new targets are important for improving our treatment of prostate cancer. Here we describe molecular characterization and drug testing in a panel of 20 prostate cancer cell lines. The cell lines cluster into distinct subsets based on RNA expression, which is largely driven by functional Androgen Receptor (AR) expression. *KLK3*, the AR-responsive gene that encodes prostate specific antigen, shows the greatest variability in expression across the cell line panel. Other common prostate cancer associated genes such as *TMPRSS2* and *ERG* show similar expression patterns. Copy number analysis demonstrates that many of the most commonly gained (including regions containing *TERC* and *MYC*) and lost regions (including regions containing *TP53* and PTEN) that were identified in patient samples by the TCGA are mirrored in the prostate cancer cell lines. Assessment of response to the anti-androgen enzalutamide shows a distinct separation of responders and non-responders, predominantly related to status of wild-type AR. Surprisingly, several AR-null lines responded to enzalutamide. These AR-null, enzalutamide-responsive cells were characterized by high levels of expression of glucocorticoid receptor (GR) encoded by *NR3C1*. Treatment of these cells with the anti-GR agent mifepristone showed that they were more sensitive to this drug than enzalutamide, as were several of the enzalutamide non-responsive lines. This is consistent with several recent reports that suggest that GR expression is an alternative signaling mechanism that can bypass AR blockade. This study reinforces the utility of large cell line panels for the study of cancer and identifies several cell lines that represent ideal models to study AR-null cells that have upregulated GR to sustain growth.

## Introduction

Prostate cancer is one of the most commonly diagnosed and leading causes of cancer related death in North American men^1^. Prostate cancer is largely driven by androgens acting through the androgen receptor to give rise to proliferative and invasive cells^2,3^. As a result, therapies aimed at inhibiting the activity of the androgen receptor have remained the primary treatment modality for men with prostate cancer for the past eighty years^4,5^.

Recent advances in anti-androgen therapy have seen the introduction of more potent AR inhibitors such as enzalutamide^6,7^ apalutamide^8,9^, and darolutamide^10^. These second generation, non-steroidal anti-androgens (NSAA) antagonize AR by tightly binding the receptor and preventing its translocation to the nucleus. Clinical trials have demonstrated strong efficacy of these NSAA, improving time to progression and extending overall survival. Androgen synthesis inhibitors, such as abiraterone^11,12^, work by inhibition of the metabolic machinery that produces androgens, and have also shown significant efficacy in patients. Unfortunately, none of these drugs are curative in patients with advanced metastatic disease, as resistance will eventually develop leading to progression^2,5^. The mechanisms by which resistance occurs remains an active area of study, with mutations or splice variants in AR^13–15^, loss of AR and activation of AR-independent bypass mechanisms^16^, glucocorticoid receptor (GR) activation^17,18^, activation of other signaling pathways^19^, and metabolic changes all implicated in resistance^19^.

Effective study of NSAA resistance requires strong model systems with which to study prostate cancer in response to therapy. Our group has previously utilized large panels of breast and pancreatic cancer cell lines to gain insight into the behavior and drug response of breast and pancreatic cancers^20–22^. We have now collected a panel of 20 prostate cancer cell lines, which represents one of the largest panels of prostate cancer cell lines available to the research community. We had three initial research objectives that we wished to complete. First, we wanted to describe the characteristics of the cell line panel at the molecular level, including expression and copy number analysis and assessment of key proteins such as AR and ERG. Second, we wanted to determine the enzalutamide response in these cells. Our finding with enzalutamide response led us the third area of research, regarding the subset of samples that showed de novo over-expression of GR and estrogen receptor (ER). These studies have identified prostate cancer cell lines in which GR over-expression may compensate for the loss of AR. Furthermore, we show that a targeted therapy against GR is effective in inhibiting the growth of these cells. These results suggest that GR-targeting in prostate cancer may represent a novel therapeutic approach in men with resistance to AR-targeted drugs and identifies model cell lines with which to study these agents in prostate cancer.

## Results

We sought to establish a panel of prostate cancer cell lines that could capture some of the clinical heterogeneity observed in prostate cancers. The full panel of cell lines that we obtained including the provider and growth media conditions are shown in Table 1. Six of the cell lines were derived from primary tumors, seven originated from lymph node metastases, two were from brain metastases, and five were from bone metastases. Multiple lines included in the panel are subclones derived from parental lines that were selected for different properties including altered growth in androgen deprived media or increased metastatic potential in xenografts. Our initial step was to characterize the molecular features of the prostate cancer cell lines in the panel to determine their similarities to prostate tumors. We performed RNAseq on the samples to derive expression data and ran SNP6.0 microarrays for copy number analysis, combined with Western blotting of ERG and AR proteins.

**Table 1:** Prostate cell line panel listing provider, growth conditions, and source of tumor used to establish the cell line. Cell lines from ATCC and UCSF can be obtained commercially.

Name	Source	Culture conditions	Patient source
22Rv1	ATCC	RPMI + 10%FBS	Primary, xenograft of CWR22R-2152
CA-HPV-10	ATCC	K-SFM + 0.05 mg/ml BPE + 5 ng/ml EGF	Primary prostate transformed with HPV18
CWR-R1	Elizabeth Wilson, UNC	DMEM + 10%FBS + additives	Primary, xenograft of CWR22R
DU145	UCSF culture facility	Eagles MEM + 10%FBS	Brain metastasis
DuCaP	Mattias Nees, VTT	RPMI + 10%FBS	Brain met; derived from same patient as VCaP
HH870	Hoag Hospital	RPMI + 10%FBS	Primary prostate cancer
IGR-CaP1	Anne Chauchereau, IGR	RPMI + 10%FBS	Primary prostate cancer
LAPC4	Joshi Alumkal OHSU	DMEM + 10% FBS + 1 nM R1881	Lymph node metastatis, xenograft
LNCaP	UCSF culture facility	RPMI + 10%FBS	Lymph node metastasis
LNCAP-19	Karin Welin, Gothenberg	RPMI + 10%FBS (charcoal stripped)	Derivative of LNCaP
LNCaP-C4	UCSF culture facility	RPMI + 10%FBS	Derivative of LNCaP
LNCaP-C4-2	UCSF culture facility	RPMI + 10%FBS	Derivative of LNCaP
LNCaP-RF	Donald Tindall, Mayo Clinic	RPMI + 10%FBS	Derivative of LNCaP
MDAPCa1	Mattias Nees, VTT	RPMI + 10%FBS	Bone metastasis; aka ARCaP
MDAPCa2b	ATCC	F12K + 20%FBS + additives	Bone metastasis
NCI-H660	ATCC	RPMI + 7.5%FBS	Lymph node metastasis; small cell cancer
PC3	UCSF culture facility	F12K + 10%FBS	Bone metastasis
PC346C	W. van Weerden, Erasmus	DMEM/F12 + 2%FBS + additives	Primary prostate cancer
PC-3 M	Joshi Alumkal OHSU	RPMI + 10%FBS	Derivative of PC3
VCaP	ATCC	DMEM + 10%FBS	Bone metastasis

### Expression analysis of prostate cell lines

**Figure 1:**
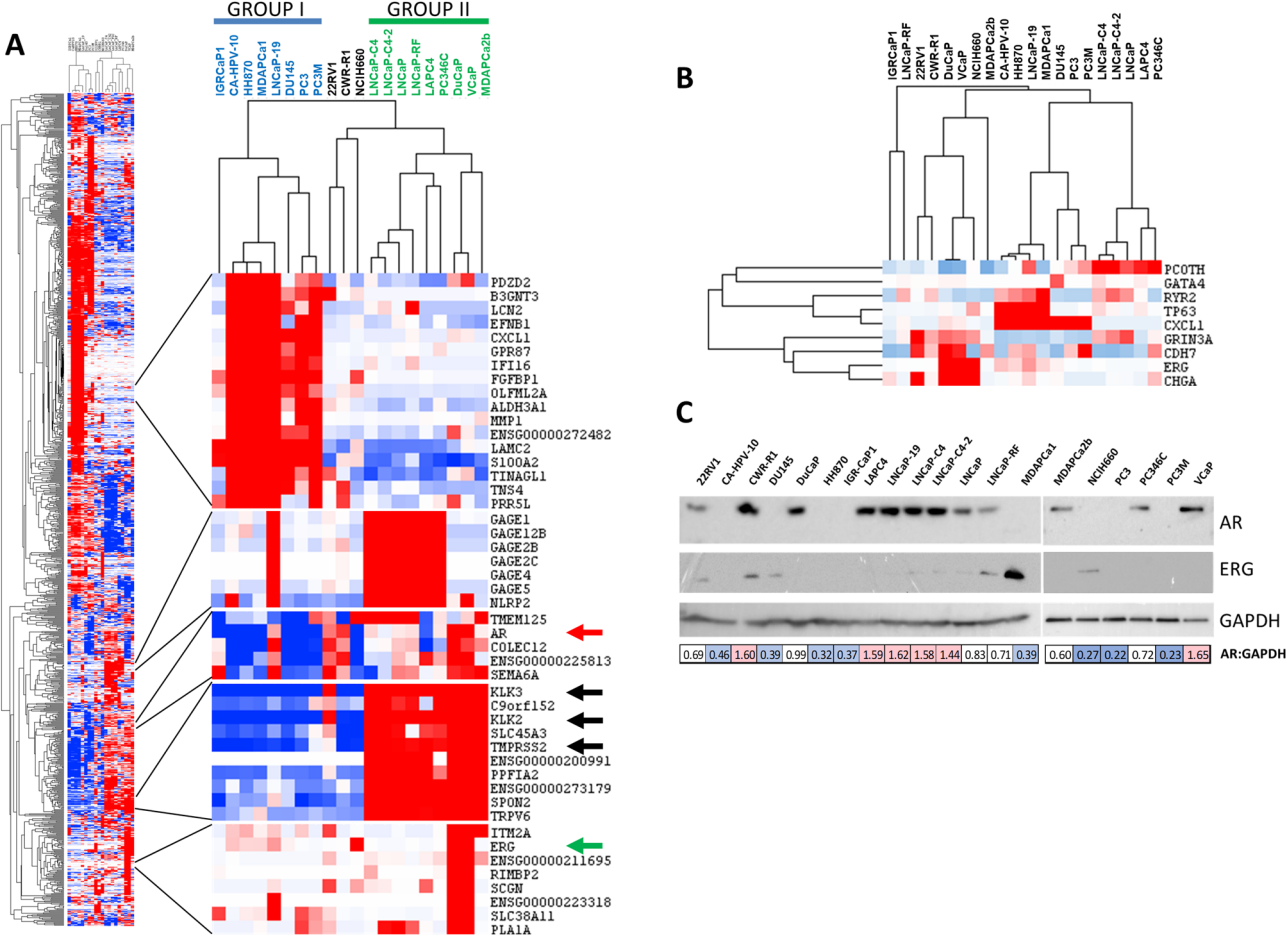
Expression of variable and selected genes and proteins in prostate cancer cell lines. (**A**) Hierarchical clustering of cell lines and the thousand most variable genes reveals distinct clusters. Several of the clusters are directly related to AR function and activity. PSA (*KLK3*) is the most variably expressed gene in the panel, but only shows weak correlation with *AR* expression. Similarly, *TMPRSS2* and *ERG* also show weak associations with *AR* RNA expression. An additional cluster of testes antigen genes is strongly expressed in LNCaP cells and derivatives, as well as several additional cell lines. (**B**) Clustering of cell lines mirrors clustering prostate tumors using genes identified by the TCGA prostate cancer. The clustering of the genes is also highly similar, although *CHGA* clusters with different genes in our data set. Furthermore, expression of *PCOTH* and *GATA4* are extremely low in the cell lines, suggesting that their expression may be confined to stromal cells in vivo. (**C**) Western blot analysis of AR and ERG expression in the prostate cancer cell line panel. Cropped images highlighting the bands specific to AR and ERG are shown. Relative AR:GAPDH expression levels are shown below, color coded by low (blue), moderate (white), and high (red) AR:GAPDH Full, unaltered blots are available in supplementary Fig. S1.

For the expression analyses, we started by using unsupervised hierarchical clustering with the top 1000 most variably expressed genes to cluster the genes and cell lines (Fig. 1A). The cell lines organized into two major clusters that roughly corresponded to their functional AR status (group I and II in Fig. 1A). Cell lines in group I had low levels of expression of *AR* and target genes like *KLK3* and *TMPRSS2*, while those in group II had high levels of expression of these genes. There was also a minor cluster consisting of the cell line NCI-H660, which was derived from a small cell cancer of the prostate, and the two related cell lines CWR-R1 and 22Rv1, which were both derived from the same patient and were initially grown as xenografts in immune-deficient mice (Fig. 1A).

The gene with the highest standard deviation in expression across the entire panel of 20 cell lines was *KLK3*, which encodes prostate specific antigen (PSA). The related kallikrein gene *KLK2* had expression that was highly correlated with *KLK3*, as did *TMPRSS2*, one of the genes involved in the TMPRSS2:ERG translocation that is common in prostate cancer. All three genes are indicated by black arrows in Fig. 1A. Despite the fact that AR is thought to be the driver of PSA expression in prostate cancer, expression of *AR* was not highly correlated with expression of *KLK3* (see red arrow, Fig. 1A). For example, the cell lines CWR-R1 and LNCaP-19 both showed elevated levels of *AR* expression, but low levels of *KLK3*, while LNCaP-C4-2 and LAPC4 cell lines had low levels of *AR* expression but high levels of *KLK3*. Finally, expression of *ERG* (see green arrow, Fig. 1A) was also not highly correlated with expression of *TMPRSS2*, which is unexpected since these genes are fused in a high percentage of prostate cancers.

Other gene clusters were also evident in the cell lines. One of the most prominent clusters was related to expression of the cancer/testis family of G-antigens (GAGE proteins; see Fig. 1A). Although most of the expression of the *GAGE* genes was found in LNCaP-derived cell lines, there was also strong expression of these genes in LAPC4 and PC-346C cells, suggesting that some prostate cancers may express these antigens. Other cancer/testis antigens such as *PAGE1* and *NXF2* (cancer/testis antigen 39) were also found associated with this gene cluster.

We performed gene set enrichment analysis (GSEA) on the samples in the two major clusters to determine what biological hallmarks distinguished the groups. Although some of the lines are subclones of parental lines, we used them all in the GSEA due to the small sample numbers. There were 31 gene sets that were upregulated with a false discovery rate (FDR) q-value less than 0.25 in the first group (supplementary Table S1A). This group, which had low AR gene expression, had significant enrichment of hallmarks of early and late estrogen response. In contrast, there were 7 hallmarks that were upregulated in the second class of cell lines, including androgen response (supplementary Table S1B). However, none of these were significant after FWER correction.

The primary TCGA study on prostate cancer identified distinct subsets of prostate cancer^23^, but most were driven by specific genomic, mutational, or epigenomic alterations. However, the TCGA paper did highlight a subset of 9 genes that most strongly associated with these different subsets at the RNA level. In our data set, expression of *TP63*, *CXCL1*, and *CDH7* were found clustered together in one set of samples that was characterized by low level of AR, while *ERG* and *CHGA* clustered together in a second set of samples that had higher levels of AR expression. The other four genes were not amongst the top 1000 most variably expressed genes. We also examined the TCGA defined genes on their own (Fig. 1B). These genes separated the cell lines into three subsets, as was seen in the TCGA study. Most of the gene clustering was the same in the cell lines as in the TCGA patient samples, although *CHGA* clustered with *ERG*, *CDH7* and *GRIN3A* in our data set instead of with *CXCL1* and *TP63* as was seen in TCGA. Expression of *PCOTH* and *GATA4* were detectable but extremely low in the prostate cell lines. This suggests that these genes are predominantly expressed in non-epithelial prostate cells associated with the tumor, they are expressed in a subset of prostate tumors not represented by current cell lines in our panel, or that expression is lost during adaptation to culture conditions.

We also examined the expression of AR and ERG at the protein level by western blotting (Fig. 1C). AR expression was variable across the cell line panel, but was strongly correlated with AR expression at the RNA level. Every cell line with high levels of AR gene expression had detectable AR expression at the protein level, including 22rv1, CWR-R1, and MDAPCa2b, all of which have splice variants of AR. Protein and RNA expression of ERG were not strongly correlated, with ERG protein expressed most strongly in 22rv1, CWR-R1, DU145, LNCaP-RF, MDAPca-1, and NCI-H660 and with detectable but lower levels in the other LNCaP cell lines.

### Copy number analyses of prostate lines

**Figure 2:**
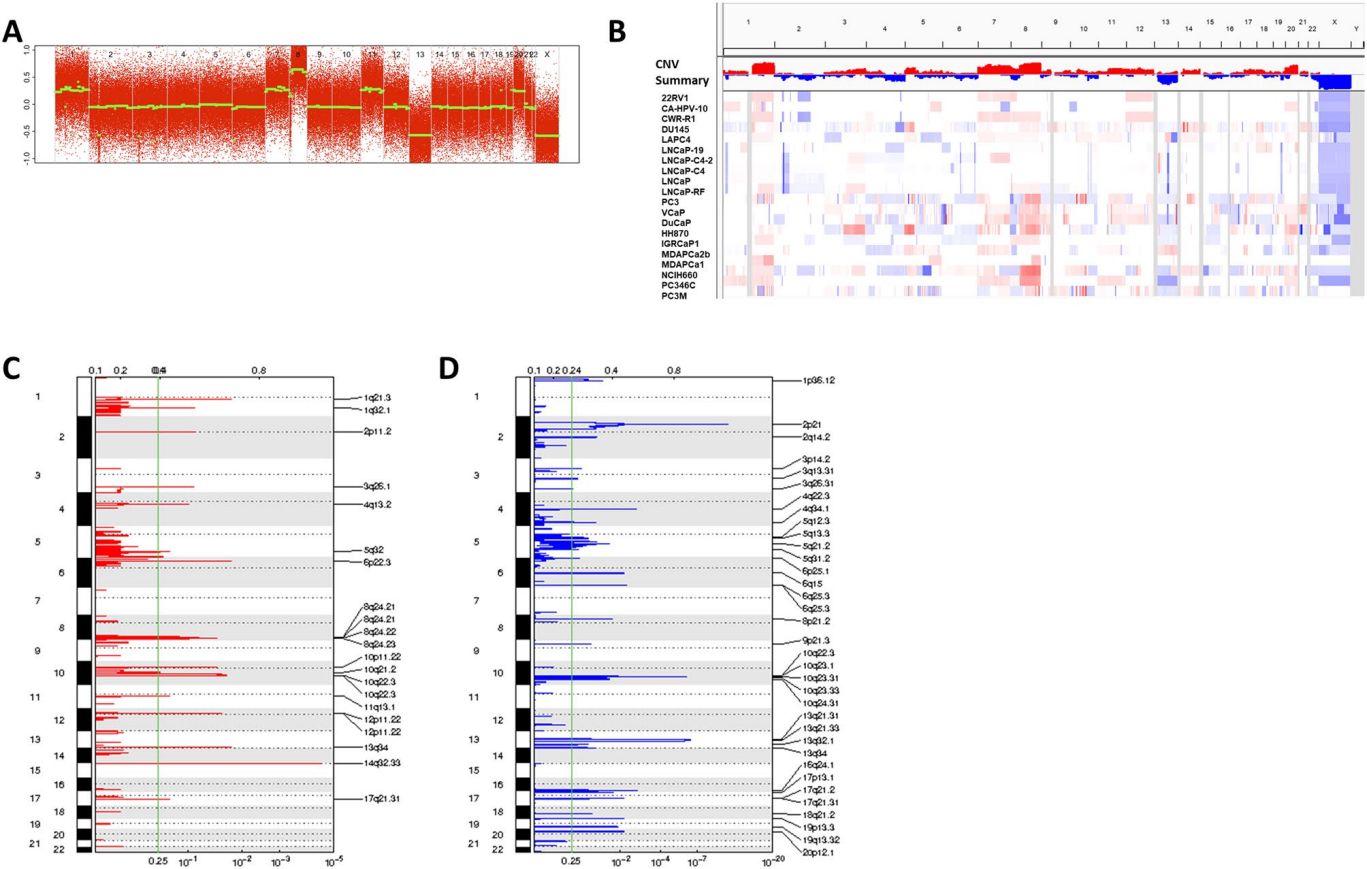
Copy number analysis of prostate cancer cell lines. (**A**) Example of ASCAT copy number estimates (green line) for the prostate cancer cell line PC346C demonstrating regions of gain and loss. (**B**) IGV view of copy number changes in individual cell lines and the average copy number changes in the entire cell line panel. (**C, D**) GISTIC analysis of recurrent regions of copy number gain (**C**) and loss (**D**). Many of the recurrent regions of copy number alterations observed in the cell lines are the same as those seen in prostate cancer specimens as defined by the TCGA.

Next, we examined copy number changes in the cell line panel using SNP6.0 microarray chips. We used the Allele Specific Copy Number Analysis of Tumors (ASCAT) method to identify copy number alterations. An example of an ASCAT segmented cell line sample is shown in Fig. 2A. We used the LogR measure of total signal intensity as the quantification for genomic copy number level for each sample, which was then loaded into the Integrated Genome Viewer (IGV) for visualization and analysis (Fig. 2B). The most commonly altered chromosomal regions were gains of 1q (75% of samples), 7 (65%), 8 (75%), and 20 (60%), and losses of 2q (40%), 4q (45%), 8p (35%), and 13q (70%).

We next used GISTIC (Genomic Identification of Significant Targets in Cancer) 2.0^24^ to identify significant regions of gain (Fig. 2C) and loss (Fig. 2D) in the prostate cancer cell line samples, which were then compared to the alterations identified in the TCGA study. We found that several of the most commonly gained regions in the prostate cell lines were closely related to those identified in patient samples by the TCGA project. These included a region on 3q26.1-3q26.2, where *TERC* is located, which showed focal gain in the cell lines LNCaP-C4-2, LNCaP-RF, DuCaP, NCIH660, and in particular, HH870, where the largest level of gain was observed. Similarly, 4q13.2–3 amplification peaks were in common between the cell lines (DuCap, VCap, PC3M, and PC3) and tumors. In contrast to the 4q13.3 peak in tumors, the 4q13.2 region was the peak in the cell lines, due to the presence of small focal amplifications in 4q13.2 in 22Rv1 and CWR-R1, which contains the testosterone metabolizing genes *UGT2B17* and *UGSTB215*. In both the prostate cell lines and tumor samples, 8q24.21, which contains the *MYC* gene, were also a major amplification peak. A final significantly amplified region in common was 14q.32.33. We also observed several regions that were significant in the cell lines that were not identified in the TCGA tumor samples. Regions that showed significant gains in only the cell lines included 11q13.1, 12p11.22 and 17q21.31.

We also examined regions that showed significant focal losses by GISTIC 2.0 (Fig. 2D). Common regions with copy number loss between the cell lines and TCGA prostate tumors included 3p13, 5q13, 8p21, 10q23 (containing PTEN), multiple sites on 13q (containing *RB*), 16q24.1, and 17q21 (containing *TP53*). Unique regions of loss in the cell lines included 1p36, 2p21, 4q22, 6q25, 9p21.3 (containing CDKN2A).

### Enzalutamide drug screen and gene expression associations with response

**Figure 3:**
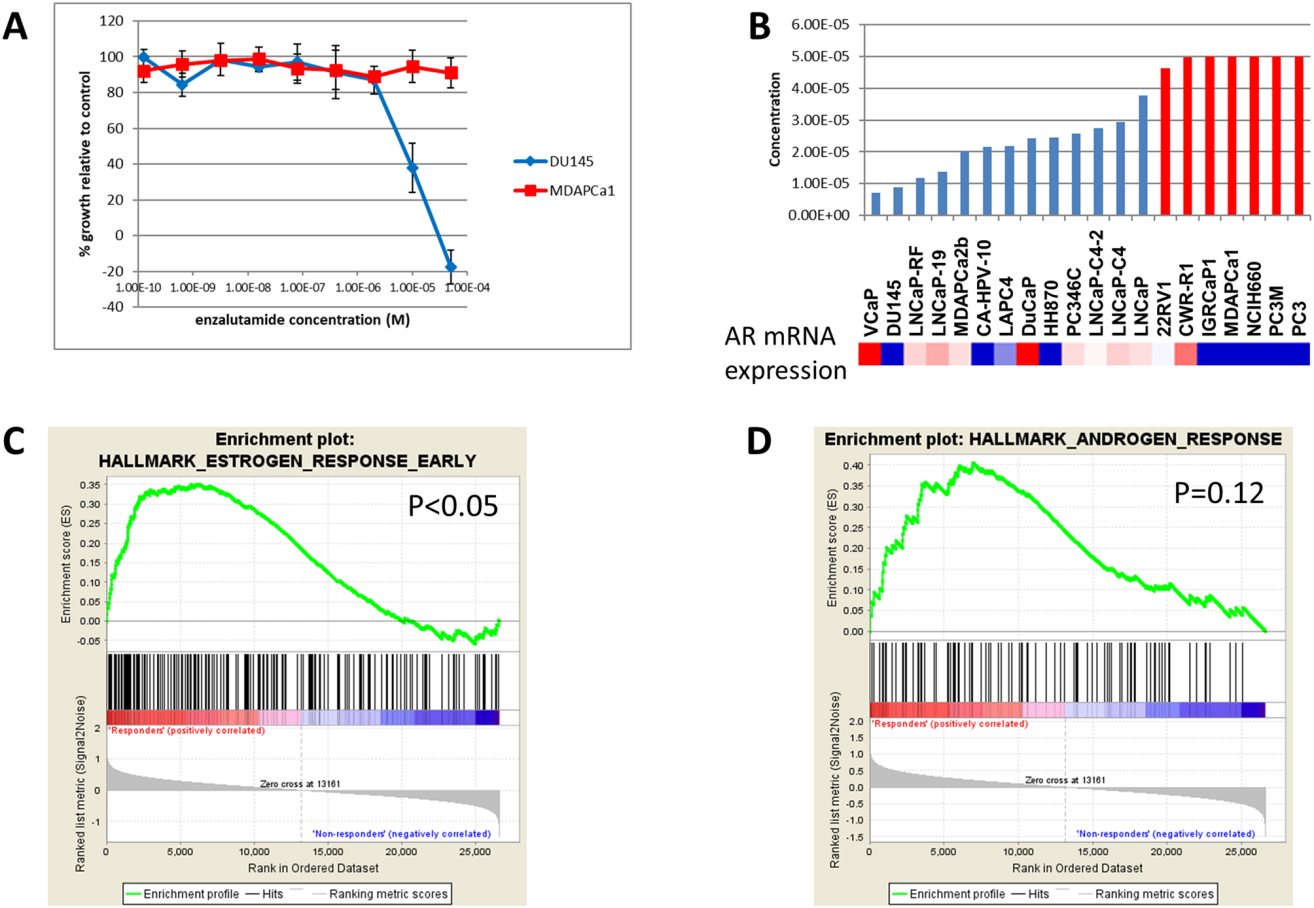
Enzalutamide response in the prostate cancer cell line panel. (**A**) Example dose response curves for a responsive (blue, DU145) and non-responsive (red, MDAPCa1) prostate cancer cell line. Error bars are +/− standard deviation of triplicate measurements. (**B**) GI50 values for each of the cell lines divides the samples into responsive (blue) and non-responsive (red) clusters. Lower bars represent lower doses of drug required to inhibit growth by 50%. For non-responsive lines, GI50 values were set to maximal dose tested. (**C,D**) GSEA plots for Estrogen and Androgen response elements show differences between enzalutamide responders and non-responders. The association is significant for ER associations (*p* < 0.05) but fails to reach significance for AR (*p* = 0.12).

We next performed drug screens on the cell line panel using enzalutamide. Consistent with previous reports, and similar to other hormone targeting agents in vitro, we found that the response as measured by GI50 (dose required to inhibit growth by 50%) were in the µM range (Fig. 3A). The response largely tracked with AR status (Fig. 3B), where responsive cells had high levels of AR protein expression and AR null cells (no detectable AR protein) were non-responsive. Similarly, cells with splice variants in AR (22Rv1, CWR-R1, and MDAPCa2b) were more resistant to enzalutamide. Surprisingly, there were several cell lines that were null for AR expression by both expression and Western analysis that were responsive to enzalutamide. This included the cell lines DU145, CA-HPV-10, and HH870.

We performed GSEA comparing various subsets of responders to non-responders. Our initial GSEA comparing all responders (blue samples in Fig. 3B) to non-responders (red samples in Fig. 3B) identified only three hallmarks that were significantly enriched prior to multiple comparison corrections in responders (late ER response, adipogenesis, and xenobiotic metabolism; see example in Fig. 3C). The hallmark for androgen receptor was borderline significant, with a nominal *p* value of 0.12 (Fig. 3D). None of these hallmarks were significant following False Discovery Rate (FDR) or Family-Wise Error Rate (FWER) corrections. We also compared just the AR positive responder cells to the non-responder cell lines, and found just two hallmarks that were significant at the nominal *p* value level (xenobiotic metabolism and reactive oxygen species pathway). Neither of these were significant after FDR or FWER corrections. Finally, we compared the AR-null responders to non-responders, and found that there were no significant hallmarks enriched in these cells, even at nominal *p* value levels.

### Potential targets of enzalutamide in AR null cell lines

**Figure 4:**
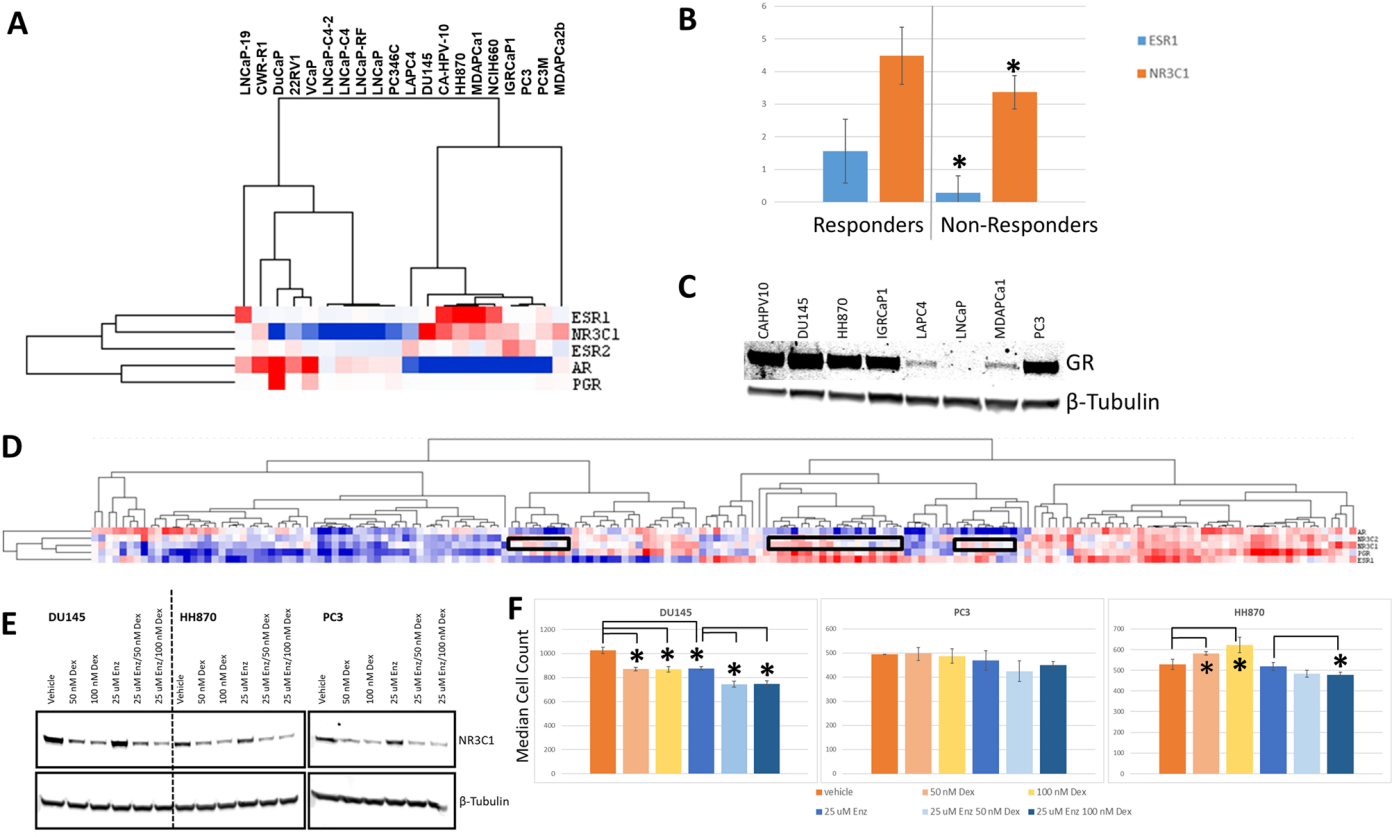
Expression of other NR3 family members in prostate lines and assessment of GR as a potential target in prostate cancer cell lines. (**A**) Expression of NR3 nuclear receptors in the prostate cancer cell line. *NR3C1* expression is largely inversely correlated with *AR* expression. (**B**) Expression of *ESR1* (blue) or *NR3C1* (orange) in AR-null responders (left) or non-responders (right) to enzalutamide, showing that the expression of both of these family members is significantly higher in the cell lines that responded to enzalutamide (*p* < 0.05). (**C**) Western blot analysis of glucocorticoid receptor in a subset of the prostate cell lines. Note expression was positive in all of the lines tested except for the AR positive cell line LNCaP. Full, unaltered blots are available in supplementary Fig. S1. (**D**) Expression of NR3 family members in the TCGA panel of prostate cell lines. Distinct clusters are evident, including tumors that lack expression of *AR* but show strong expression of *NR3C1* (boxes). (**E**) Treatment of cells with dexamethasone (50 or 100 nM) results in down-regulation of GR protein, including in the presence of enzalutamide, in DU145, HH870, and PC3 cells. (**F**) Dexamethasone treatment significantly impacts cell growth and enzalutamide response in DU145 and HH870 cells (significant differences marked with bars to show comparisons tested and * to indicate significance). Treatment with either 50 or 100 nM dexamethasone inhibits the growth of DU145 cells (peach and yellow colored bars, *p* < 0.001), similar to the treatment with enzalutamide (blue bar, *p* < 0.001). Addition of dexamethasone to enzalutamide (light blue and dark blue bars) result in significant growth inhibition compared to enzalutamide alone (*p* < 0.001 in both cases). In HH870 cells, dexamethasone treatment (peach and yellow colored bars, *p* < 0.005) enhances cell growth compared to control. Treatment with 100 nM dexamethasone (dark blue bar) results in significant growth inhibition compared to enzalutamide alone (*p* < 0.05). Treatment with 50 nM dexamethasone plus enzalutamide also shows a trend towards enhancing response (*p* = 0.069).

The response of several AR null cell lines to enzalutamide was surprising. Since ER response appeared in the GSEA comparison between responders and non-responders, we wondered if other members of the type III nuclear receptor (NR3) subfamily might be responding to enzalutamide at the high levels that are required to see activity in short-term in vitro assays. We hypothesized that at high concentrations, enzalutamide may bind to these structurally-related proteins. We did observe that both the estrogen receptor (ER, encoded by *ESR1*) and glucocorticoid receptor (GR, encoded by *NR3C1*) were inversely expressed compared to AR (Fig. 4A), and that several of the AR null lines (DU145, CA-HPV-10, and HH870) that responded to enzalutamide had high levels of *NR3C1* expression. We compared the average expression of *NR3C1* and *ESR1* in the AR null lines that responded to enzalutamide versus the AR null lines that did not respond to enzalutamide and found that the average expression of the genes was significantly lower in the non-responder lines (Fig. 4B). Progesterone Receptor (PGR) did not show an association between enzalutamide response and gene expression, and gene expression of *PGR* was much lower than either *NR3C1* or *ESR1*. Mineralcorticoid receptor was not expressed at significant levels in any of the samples. These gene expression, GSEA associations, and previous reports of GR involvement in castration-resistant prostate cancer indicated that other NR3 receptors and the networks controlled by them might be operational in some of these cell lines. We performed Western blot analysis for expression of GR in a subset of the cell lines, and found that all of the lines tested except for LNCaP expressed detectable levels of GR (Fig. 4C). Furthermore, the AR-null lines that responded to enzalutamide had the highest levels of GR (DU145, CA-HPV-10, and HH870). Surprisingly, when we examined expression of GR in TCGA samples, we found that the average expression of *NR3C1* was higher than that of *AR*. There was a large cluster of samples that had high co-expression of *AR* and *NR3C1* (Fig. 4D), suggesting there is validity to co-targeting AR and GR in patients. There were also several subclusters of samples that had low *AR* expression but higher than average *NR3C1* expression levels (Fig. 4D), consistent with reports that GR may be an alternative pathway for AR related signaling in castration resistant tumors^17^. *ESR1* expression largely tracked with *AR* expression, although there were distinct clusters with inverse expression levels (Fig. 4D).

These data suggested that enzalutamide might be binding to GR and impacting response. To test this, we examined enzalutamide response in three prostate cancer cell lines, PC3, HH870 and DU145, which express moderate to high levels of GR protein, in the presence of dexamethasone. Dexamethasone treatment has been reported to reduce expression of GR^25^, and thus we reasoned that dexamethasone treatment might sensitize cells to enzalutamide, since there could be saturation of the target with the same amount of drug but less protein. As expected, treatment with dexamethasone decreased GR expression in all three prostate cancer cell lines at both 50 and 100 nM doses (Fig. 4E). We found that in PC3 cells treatment with 50 or 100 nM dexamethasone did not significantly impact the survival of cells. Dexamethasone at both doses resulted in a modest but significant growth inhibition in DU145, similar to the inhibition of growth caused by enzalutamide at 25 µM (see Fig. 4F), while the same treatments in HH870 resulted in a modest but significant growth enhancement (Fig. 4F). As expected, enzalutamide alone showed significant growth inhibition in DU145 cells at 25 µM, but had limited impact on PC3 and HH870 at that dose. However, the combination of enzalutamide in the presence of dexamethasone resulted in a significant inhibition of growth in DU145 (at both doses of dexamethasone) and HH870 (at 100 nM dexamethasone). PC3 cells showed a small decrease in cell number with dexamethasone treatment, but this did not reach signficance.

### Testing of mifepristone

**Figure 5:**
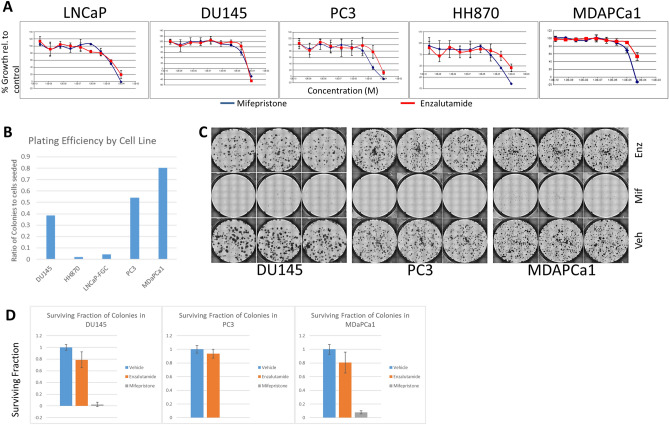
Growth of prostate cancer cell lines treated with enzalutamide versus mifepristone. (**A**) Response of cell lines to enzalutamide (red) or mifepristone (blue) shows that mifepristone is more efficacious in several of the cell lines. (**B**) Assessment of plating efficiency of cell lines used in clonogenic assay testing mifepristone versus enzalutamide response. (**C**) Crystal violet stain for colony formation in DU145, PC3, and MDAPCa1 cells. 25 µM enzalutamide has minimal impact on colony formation in these cells compared to vehicle controls, whereas an equivalent dose of mifepristone almost completely inhibits the ability of these cells to form colonies. (**D**) Quantification of the response to mifepristone and enzalutamide in three AR null cell lines (DU145, PC3, and MDAPrCA1) shows that mifepristone inhibits colony growth more robustly than enzalutamide (at 25 µM each).

We decided to further investigate the potential role for GR in AR-resistant prostate cell lines, since it had previously been implicated in castrate resistant disease, it had higher levels of expression than *ESR1* in the prostate cancer cell lines, showed an inverse correlation in expression with AR in both the cell lines and patient samples in the TCGA data set, and treatment with dexamethasone altered enzalutamide response in 2 of our 3 AR-null cell lines. We treated selected prostate cancer cell lines with the PGR/GR antagonist mifepristone to determine the ability of this drugs to inhibit the growth of prostate cancer cell lines. We used the cell lines LNCaP (as a control) and HH870, PC3, MDAPCa1, and DU145 (all of which expressed moderate to high levels of GR; see Fig. 4A) to test response to mifepristone and enzalutamide at nine different concentrations (fivefold dilutions), with a highest dose of 100 µM (see Fig. 5A). We used GR50^26^, a metric highly related to GI50, to measure response in the cells. Mifepristone showed equivalent (DU145, LNCaP) or better (HH870, PC3, MDAPCa1) growth inhibition compared to enzalutamide in every line tested. The GR50 values for mifepristone were fivefold lower for HH870, PC3, and MDAPCa1 than for enzalutamide. We summarized the expression of AR, GR, and the response to enzalutamide and mifepristone in Table 2. These data suggest that several prostate cell lines were dependent on GR activity for growth and survival.

**Table 2:** Expression of androgen receptor and glucocorticoid receptor transcripts and proteins and their response to enzalutamide and mifepristone in prostate cancer cell lines.

Name	AR mRNA	AR protein	GR mRNA	GR protein	Enzalutamide response	Mifepristone response
22Rv1	High	Pos^a^	Low	ND	Resistant	ND
CA-HPV-10	Low	Neg	High	High	Sensitive	ND
CWR-R1	High	pos^a^	High	ND	Resistant	ND
DU145	Low	Neg	High	High	Sensitive	Sensitive
DuCaP	High	Pos	Low	ND	Sensitive	ND
HH870	Low	Neg	High	High	Sensitive	Sensitive
IGR-CaP1	Low	Neg	High	High	Resistant	ND
LAPC4	Low	Pos	Low	Mod	Sensitive	ND
LNCaP	Moderate	Pos	Low	Low	Sensitive	Sensitive?
LNCAP-19	High	Pos	Mod	ND	Sensitive	ND
LNCaP-C4	Moderate	Pos	Low	ND	Sensitive	ND
LNCaP-C4-2	Moderate	Pos	Low	ND	Sensitive	ND
LNCaP-RF	Moderate	Pos	Low	ND	Sensitive	ND
MDAPCa1	Low	Neg	High	mod	Resistant	Sensitive
MDAPCa2b	Moderate	Pos	High	ND	Sensitive	ND
NCI-H660	Low	Neg	High	ND	Resistant	ND
PC3	Low	Neg	Mod	high	Resistant	Sensitive
PC346C	Moderate	Pos	Low	ND	Sensitive	ND
PC-3 M	Low	Neg	High	ND	Resistant	ND
VCaP	High	Pos	Low	ND	Sensitive	ND

^a^CWR-R1 and CA-HPV-10 express high levels of AR transcript and protein but are known to harbor splice variants that impact enzalutamide response.

Pos: positive expression; Mod: moderate expression; Neg: negative expression; *ND* not determined.

We next tested the ability of mifepristone to inhibit the growth of prostate cancer cell lines in clonogenic assays. We plated a set of 5 cell lines (LNCaP, PC3, DU145, HH870, and MDAPCa1) at low density and grew them for 7–14 days in the presence of 25 µM enzalutamide or mifepristone compared to vehicle-treated control cells. The plating efficiency of LNCaP and HH870 was low (4.3% and 1.9% respectively), so they gave limited information about drug response. The other three cell lines all had higher plating efficiencies (MDAPCa1: 80.5%; PC3: 54.1%; DU145: 38.4%), allowing assessment of drug efficacy at 7 days (Fig. 5B). Enzalutamide at 25 µM only inhibited growth in LNCaP cells (although this was difficult to assess given the low plating efficiency). In contrast, mifepristone resulted in significant inhibition of cell growth in all of the cell lines (Fig. 5C,D). We observed that mifepristone treatment resulted in reduction in the formation of colonies (0% of control in PC3, 2.5% of control in DU145, and 7.8% of control in MDAPCa1 cells). In contrast, enzalutamide treatment still resulted in colony formation at a rate of at least 75% of the control condition each of these three lines. These results show that mifepristone treatment, but not enzalutamide treatment, could inhibit the growth of these cell lines showing moderate to high levels of GR expression in the absence of AR expression.

## Discussion

Cell lines have been useful models for the study of cancer, maintaining many of genomic changes, mutations, and expression subtypes present in patient tumors^27,28^. Cell lines have proven useful for extensive drug screening studies and gene silencing studies, such as the NCI60 panel^29^, the Cancer Cell Line Encyclopedia^30^, and project Achilles^31^. However, these studies rely on the availability of large numbers of high quality cell lines. For prostate cancer, this has been particularly problematic, as it has been difficult to grow prostate cancer cells in vitro, and many prostate cancer cell lines are not representative of clinically observed prostate tumors. To that end, we obtained prostate cancer cells from both commercial and individual sources and performed in depth molecular analyses and drug screening on the twenty lines that we acquired. In terms of copy number, the prostate cancer cell lines showed gains and losses in many of the most commonly altered regions that were observed in patient samples by the TCGA. As expected, we also observed some regions that were unique to the cell lines, such as losses in 1p36, 2p21, 4q22, 6q25, and 9p21.3, and gains in 11q13.1, 12p11.22 and 17q21.31. These may harbor genes that are important for adaptation to culture conditions.

The cell lines expressed many of the hallmark genes that would be expected in prostate cancer. For example, *KLK3*, which encodes prostate specific antigen, was highly expressed in a subset of the samples (and also showed the largest standard deviation in expression across the panel). *AR* expression was not correlated strongly with *KLK3* gene expression, consistent with previous reports that show a complex relationship between *AR* and *KLK3* expression^23,32,33^. The TCGA study found that AR activity, as inferred by expression of AR target genes including *KLK3*, was not correlated with levels of AR expression, but instead varied by the molecular subtype^23^. Furthermore, c-MYC overexpression has been shown to oppose the action of AR on several AR-target genes like *KLK3*^33^, and thus cell lines that have high levels of MYC and AR might have lower levels of *KLK3* than would be expected. Finally, the presence of AR splice variants in some cell lines which may alter the function of the protein without changing *AR* expression. The cell lines CWR-R1 and 22RV-1 both express high levels of the AR splice variant, but do not express *KLK3* at a significant level.

Interestingly, we also observed that a subset of the cell lines expressed high levels of MAGE and GAGE antigens. Although this was mainly seen in LNCaP and derivatives of LNCaP, we also saw significant expression in some non-LNCaP cell lines such as LAPC4, indicating it could be a more general phenomenon. We assessed expression of GAGE genes in TCGA samples and found that ~ 10% of patients showed detectable levels of expression of *GAGE10* and *NXF2* antigens, although this was typically at lower levels than those observed in the cell lines. The presence of these antigens in LNCaP cells was recognized more than 20 years ago^34^, but their relevance remains unclear. However, these cancer/testes antigen genes remain under study as potential targets for immunotherapy (reviewed in Ref^35^).

We also examined expression and subtyping of the prostate cell lines compared to the genes and subtypes identified by the TCGA prostate study^23^. Interestingly, two of the definitive subtype specific genes found by the TCGA were not expressed above background levels in any of the prostate cancer cell lines (*PCOTH* and *GATA4*). The lack of expression of these two genes suggests one of three possibilities. First, these genes could be expressed in non-epithelial prostate cells associated with the tumor and thus are not present in the cell lines that are derived from epithelial cells. Second, the genes may be expressed in a subset of prostate tumors that are not represented by current cell lines in the panel, perhaps reflecting the difficulty in culturing cells of a specific subtype. Third, it may be that their expression is lost during adaptation to culture conditions. Immunohistochemical staining of prostate cancer tissues would be the best approach to determine if expression is confined to non-epithelial cells. Answering the other possibilities would likely require attempts to generate new cell lines with characterization of the primary tumor for comparison of expression levels of these genes.

We next tested the ability of enzalutamide to inhibit the growth of all the cell lines in the panel. The doses required to inhibit the growth of even sensitive cells were in the µM range, as has previously been reported^7^. We observed that response largely tracked with AR expression status: cell lines with high levels of wild-type AR were more responsive to enzalutamide than cell lines with low levels of AR or AR splice variants. However, we did note that some cells that were AR null were still amongst the most responsive to enzalutamide. GSEA analysis suggested that androgen and estrogen signaling were high in these cells. We hypothesized that other NR3 family receptors (glucocorticoid, progesterone, estrogen and mineralcorticoid receptors) could mimic expression of AR, and that the relatively high concentrations of enzalutamide that were used in vitro might result in targeting of other NR3 family receptors. Thus, we examined their expression in the prostate cancer cell lines. We found that *NR3C1* (glucocorticoid receptor) mRNA and GR protein was expressed at moderate to high levels in a subset of the prostate cancer cell lines. In contrast, *PGR* expression was very low according to the RNAseq data. *ESR1* expression was lower than *NR3C1* but higher than *PGR*. We tested the expression of GR at the protein level and found moderate to high levels of GR in multiple cell lines. Treatment of cells with dexamethasone lowered the expression of GR as expected^25^, resulting in increased sensitivity to enzalutamide in 2 out of the three cell lines we tested. Importantly, PC3 was the cell line that did not show any response to dexamethasone, and it was the only one of the three cell lines that did not respond at all to enzalutamide in our initial screens, suggesting that there may be alternative survival mechanisms present in these cells when enzalutamide is used. Together, these data are consistent with the possibility that GR activity may be impacted in these cell lines when treated with high doses of enzalutamide.

We thus tested the ability of the GR/PGR inhibitor mifepristone to inhibit the growth of prostate cancer cell lines compared to enzalutamide. We selected five lines for screening, with LNCaP serving as a control. The remaining four cell lines all had low levels of AR but moderate to high levels of GR. Mifepristone was highly effective against the low expressing AR lines, with GR50 values lower than those observed for enzalutamide in the same lines. Clonogenic assays demonstrated the strong ability of mifepristone to inhibit the growth of PC3, DU145, and MDAPCa1 (HH870 cells were also inhibited, but the plating efficiency was too low to be definitive). This agrees with recently published data that shows that both CWRR1 and LAPC4 as well as PC3 and DU145 prostate cells can also be inhibited by mifepristone or by knockdown of GR using inducible shRNA^36^. Interestingly, our expression profiling and Western analysis shows that LAPC4 and MDAPCa1 have moderate levels of *NR3C1*/GR expression. This suggests that even moderate levels of GR protein may be sufficient to signal efficiently, allowing escape from AR inhibition.

Multiple studies have now implicated GR signaling as an alternative method of activating growth signaling in castration-resistant prostate cancer^17,36^. Our cell line data shows that treatment of prostate cells expressing high levels of AR with mifepristone results in potent inhibition of growth. The levels of mifepristone that we used to inhibit the growth of cells in the clonogenic assay are comparable to those that are achievable in the serum of patients^37^. Our data also identifies multiple cell lines with low levels of AR expression that instead appear to use GR mediated signaling as a resistance mechanism. Importantly, other groups have demonstrated that GR upregulation can result from long-term exposure to anti-androgens such as enzalutamide or abiraterone^36,38^. However, cell lines in the study like HH870 that have low AR expression but high GR expression were established from patients prior to treatment with any anti-androgens^39^. The absence of added androgens in the growth medium suggests that up-regulation of GR could be an event that occurs during selection for cells that grow in vitro.

An important question that remains incompletely answered by our study is why enzalutamide was effective in some of the AR-null cell lines. We have shown that these lines express high levels of GR. We hypothesize that the high concentrations of enzalutamide utilized in the in vitro assays may result in binding of the drug to related NR3C family members such as GR. Surprisingly, we could not find any information about enzalutamide affinity for other NR3C family, although the ligand binding domain of GR and AR are reported to have 51% sequence identity^40^. Ideally, we would knock out GR and determine whether this alters response to enzalutamide. However, it has been shown in a recent publication using shRNA knockdown that the same prostate cancer cell lines require GR expression for viability^36^. Thus, it would require engineering a mutant GR that no longer binds to enzalutamide to demonstrate that enzalutamide is effective in these AR-null lines through off-target binding to related NR3 family members. A separate approach, which we plan to assess in follow up studies, would be to identify the proteins that bind to enzalutamide to determine if GR is bound to enzalutamide at high concentrations.

To our knowledge, this panel of prostate cancer cell lines represents one of the largest collections of molecularly characterized prostate cancer cell lines in the world. Our profiling data and molecular analyses in concert with drug screening studies strongly implicate GR-mediated signaling in cell lines that have high GR but low AR expression. Analysis of TCGA patient samples suggests the presence of patients with prostate cancer who have similar GR/AR expression profiles. In summary, these data strongly support the role for GR inhibition in patients and demonstrate the efficacy of mifepristone in suppressing the growth of AR-null, GR-expressing prostate cancer cells in vitro. Ongoing studies using mifepristone alone or in combination with enzalutamide should determine the efficacy of GR-based inhibition in patients with castration-resistant prostate cancer.

## Materials and methods

### Cell lines

We obtained cell lines from the sources indicated in Table 1. Cells were maintained at 37C in 5% CO_2_ in the media conditions recommended by the supplier (see Table 1). All cultures were maintained in antibiotic free medium to avoid interactions with drugs during testing. All cultures were regularly assessed for mycobacterial infection as described previously^41^. Cell line identity was confirmed using STR genotyping analysis (Genetica). Genotypes for all of the cell lines are listed in supplementary Table S2. Cell lines were taken from low passage, frozen stocks and maintained at subconfluent levels. Cell lines were never passaged more than 15 times.

### Protein isolation

Cells were grown on 60-mm tissue culture treated dishes to 70–90% confluence in a humidified incubator running at 37 °C/5% CO2. For harvest, dishes were placed in the biosafety cabinet on ice. Media was aspirated and cells were rinsed with sterile 1 × PBS two times. RIPA buffer (Sigma #R0278) containing protease-phosphatase inhibitor (Halt #1861281) was added. Slurry was scraped and collected into pre-chilled and labeled microtubes. Protein was then placed in the -80 °C for a minimum of 16 h. Slurries were thawed on ice then spun down at 4 °C, 15 K rpm for 10 min. The supernatant was transferred to a clean, pre-chilled and labeled microtube on ice. Protein concentration was determined using a 96-well format BCA assay (ThermoFisher Scientific #23225) with a Promega Glomax.

### Western analysis

20 µg protein samples including 4 × loading dye (Invitrogen #NP0007) w/ β-mercaptoethanol and RIPA buffer were boiled at 90 °C for 5 min. Contents were collected with a quick spin in the centrifuge. Samples were added to NuPAGE 4–12% Bis–Tris gels (Invitrogen #NP0335BOX, #NP0336BOX) and run in MOPS SDS running buffer (Invitrogen #NP0001-02) at 120 V for ~ 1 h on ice. Proteins from the gel were transferred at 30 V for 1.5 h to Immobilion membranes (Millipore #IPFL00010) in tris–glycine transfer buffer (Fisher Scientific #BP13064) containing methanol and insulated with ice water. Membranes were briefly rinsed in TBS after protein transfer, then placed in 5% BSA in TBS-T to block at room temperature for 1 h. Membranes were either probed with 1:1000 androgen receptor (GTX #62599) in 5% BSA in TBS-T or 1:2000 ERG (GTX #62386) at 4 °C overnight. Membranes were rinsed in 1 × TBS-T 3 times quickly, then 3 times for 5 min on a rocking platform at room temperature. They were then probed with 1:10,000 donkey anti-rabbit IgG-HRP conjugate (Jackson Laboratories #711–035-152) in 5% BSA in TBS-T for 45 min at room temperature on a rocking platform and rinsed as previously described. Membranes were placed on clear plastic and a chemiluminescent substrate (ThermoFisher Scientific #34080) added. Membranes were visualized on a Syngene PXi imaging system. Membranes were then rinsed and re-probed for the loading control at 1:1000 with GAPDH (Cell Signaling Technology #2118) in 5% BSA in TBS-T for 2 h room temperature, rinsed, probed with secondary and imaged as previously described.

### RNAseq

RNA was isolated from subconfluent cell lines using an RNeasy minikit (Qiagen) and submitted to the OHSU massively parallel sequencing core for RNAseq analysis. We used the Kallisto software to determine RNA expression as Fragments per Kilobase Million (FKPM). The scripts used to process the data are available at: https://github.com/danielderrick/prost_RNAseq_reprocessing. Data was visualized using standard clustering approaches^42^. Gene set enrichment analysis was performed using the online GenePattern tool from the Broad Institute and the Hallmarks gene set^43,44^. The log transformed FKPM values for the cell lines are available in supplementary Table S3.

### SNP6.0 copy number analysis

DNA was isolated from the cell lines as previously described^21^. Samples were submitted to either the Lawrence Berkeley National Laboratory HTA microarray facility or at the OHSU genome core facility for analysis on the SNP6.0 chip platform. The resulting data was process using a Python3 wrapper (https://gitlab.com/biotransistor/myascat) that ran the R based ASCAT (allele-specific copy number analysis of tumors) SNP analysis pipeline (version 2.4.4) to generate copy number data^45^. Briefly, we used 1258 SNP6 cell files, available from the HapMap project (phase3 2009–04-02), as the normal reference samples. The 13 prostate cell line samples from 2009 and 8 prostate cell line samples run in 2015 were processed in separate runs then combined. SNP6.0 cel files were transformed into the BAF and LogR illummina file format, utilizing apt-probeset-genotype and apt-probeset-summarize commands from affymetrix command line tools (version 1.18.0) and the normalize_affy_geno_cluster.pl script form PennCSV (version 1.0.3), as described in the ASCAT documentation. ASCAT was run on the BAF and LogR files to generate major minor allele track files (segemnet.txt) and PCF (piecewise constant fitting) segmented BAF and LogR track files. These track files were then transformed into seg files to be able to study the minor and major allele, the B allele frequency and the total copy number of each genomic locus tracks in the IGV genome browser (http://igv.org/). Results for PCF gamma segmentation setting 25 (ASCAT default) and 40 (PennCSV default) were produced, but all GISTIC analyses were performed on segmentation settings of 40.

### Drug response

Response to drug was assessed as described previously^21,22^, with minor modifications. Briefly, cells were plated into 96 well plates and allowed to attach overnight. The next day, cells were treated with triplicates of nine concentrations for each of three different drugs. One plate of cells was fixed, stained with DAPI, and imaged to establish cell number at time 0. Drug was left in the medium for 72 h at which point they were fixed and imaged for assessment of response. Drug response was calculated as one of two closely related metrics (earlier experiments used the GI50 metric^21,22^ while more recent experiments used the GR50 value^26^). Clonogenic assays were performed using standard methodology. Briefly, cells were plated at low density (500 cells/well) into 6 cm^2^ plates and after overnight attachment, were treated with drug or PBS as a control. Medium and drug were replenished every 2 days. Cells were fixed and stained with crystal violet 10–14 days post-plating and scanned using a STEMvision system (Stem Cell Technologies) and assessed using manual counting in Fiji^46^.

## Supplementary information

Supplementary Figure S1.

Supplementary Table S1A.

Supplementary Table S1B.

Supplementary Table S2.

Supplementary Table S3.

## Data Availability

The RNAseq data is available as a supplementary Table S3 to the manuscript. Other data is available upon request to Dr. Korkola. Sources indicated can be contacted for requests for access to specific cell lines.
