# Supplementary material for: Enzalutamide response in a panel of prostate cancer cell lines reveals a role for glucocorticoid receptor in enzalutamide resistant disease
Source: Sci Rep. 2020 Dec 10;10:21750. doi: 10.1038/s41598-020-78798-x (PMC7729982; doi:10.1038/s41598-020-78798-x)

Response to enzalutamide in a panel of molecularly characterized prostate cancer cell lines reveals a role for glucocorticoid receptor in resistant disease

Rebecca Smith[1], Moqing Liu[1], Tiera Liby[1], Nora Bayani[2], Elmar Bucher[1], Kami Chiotti[3], Daniel Derrick[1], Anne Chauchereau[4], Laura Heiser[1,5], Joshi Alumkal[6,#], Heidi Feiler[1,5], Peter Carroll[7], and James E. Korkola[1,5].

[1] Department of Biomedical Engineering, Oregon Health & Science University, Portland, OR 97201
[2] Lawrence Berkeley National Laboratories, Life Sciences Division, Berkeley, CA 94720
[3] Department of Molecular and Medical Genetics, Oregon Health & Science University, Portland, OR 97201
[4] INSERM U981, Gustave Roussy Institute, Paris-Saclay University, 94800 villejuif, France
[5] OHSU Center for Spatial Systems Biomedicine, Oregon Health & Science University, Portland, OR 97201
[6] Department of Medicine, Oregon Health & Science University, Portland, OR 97201
[7] Department of Urology, UCSF, San Francisco, CA 94158
[#]Current Address: Department of Internal Medicine, Michigan Medicine, Ann Arbor, MI 48109
Corresponding Author: James Korkola, 2730 SW Moody Ave CLSB Rm 3N018, Portland, OR 97201
Email: korkola@ohsu.edu

Supplemental Figure S1: Androgen Receptor blots for prostate cancer cell lines (corresponding to main Fig.1C).

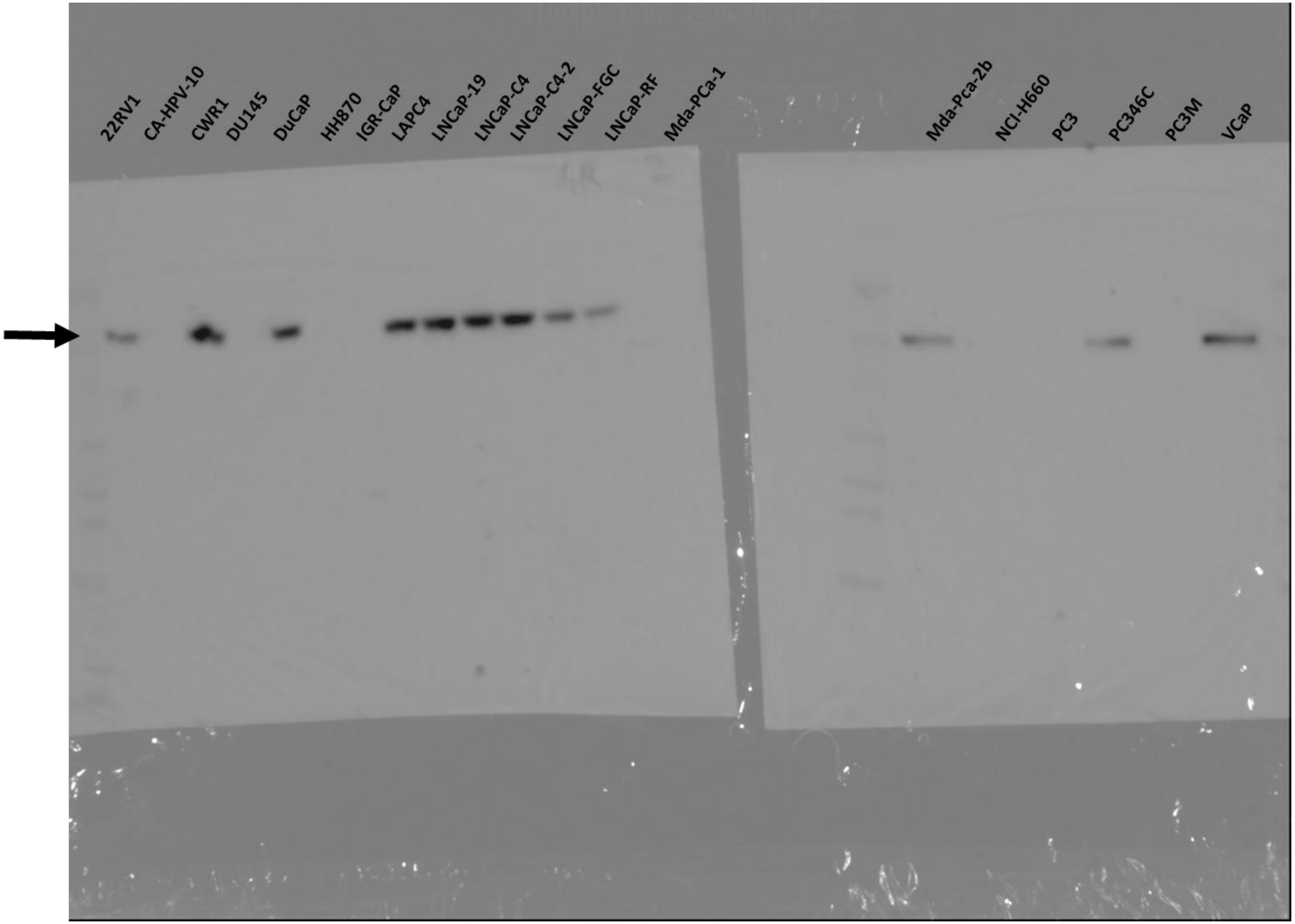

Supplemental Figure S1: ERG blots for prostate cancer cell lines (corresponding to main Fig.1C).

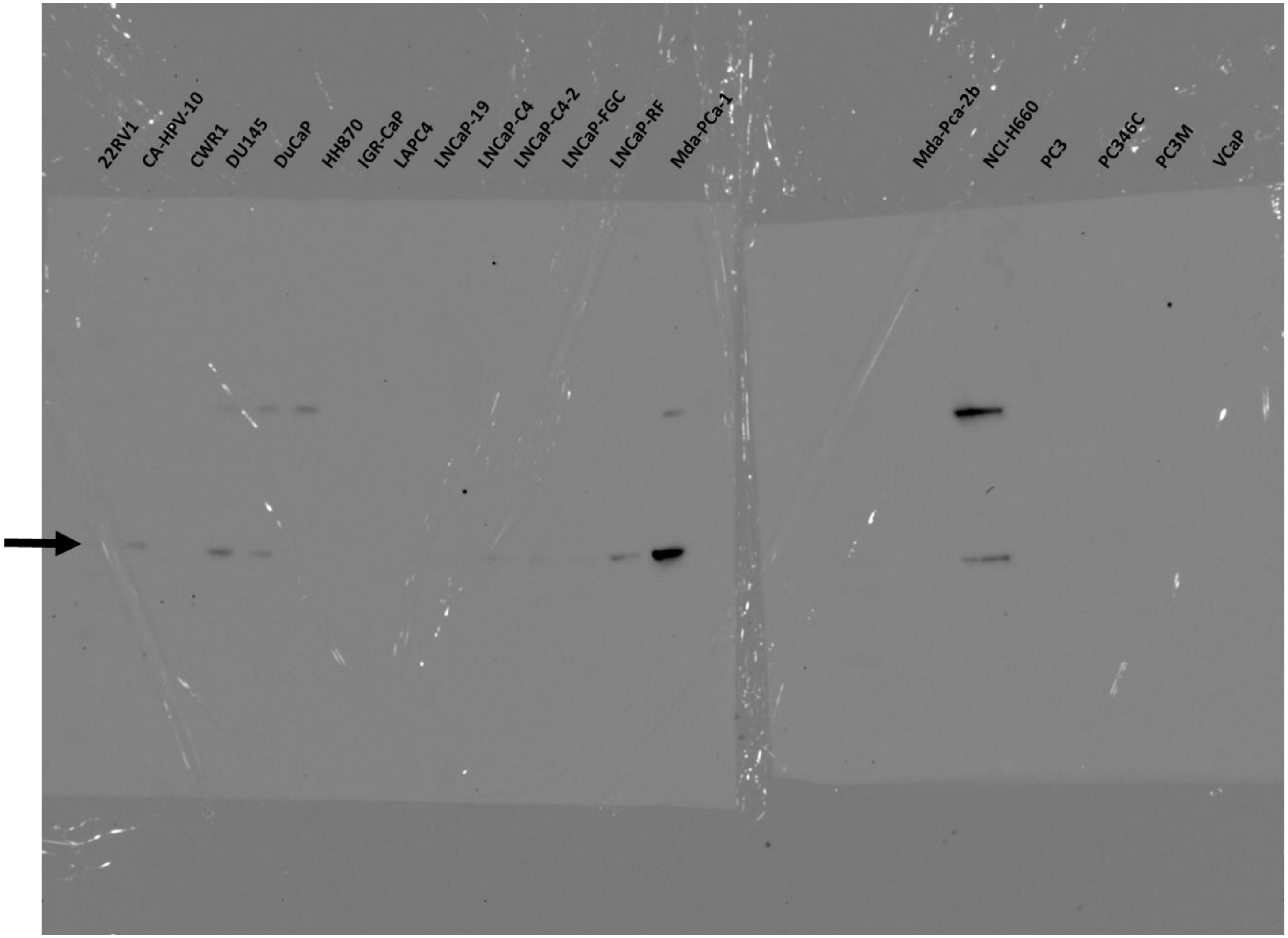

Supplemental Figure S1: GAPDH loading control blots for prostate cancer cell lines (corresponding to main Fig.1C).

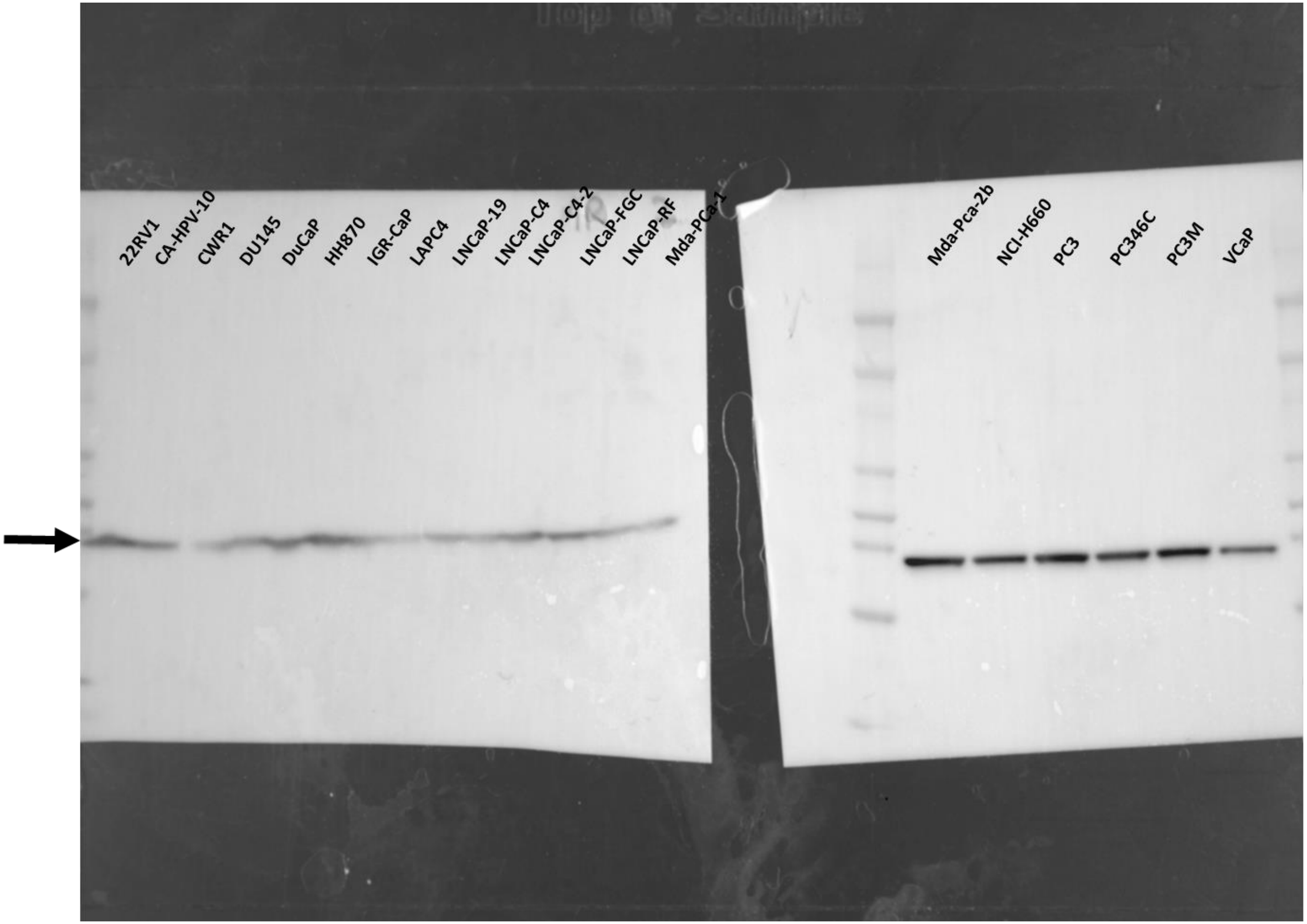

Supplemental Figure S1: GR Western blots for prostate cancer cell lines (corresponding to main figure 4C)

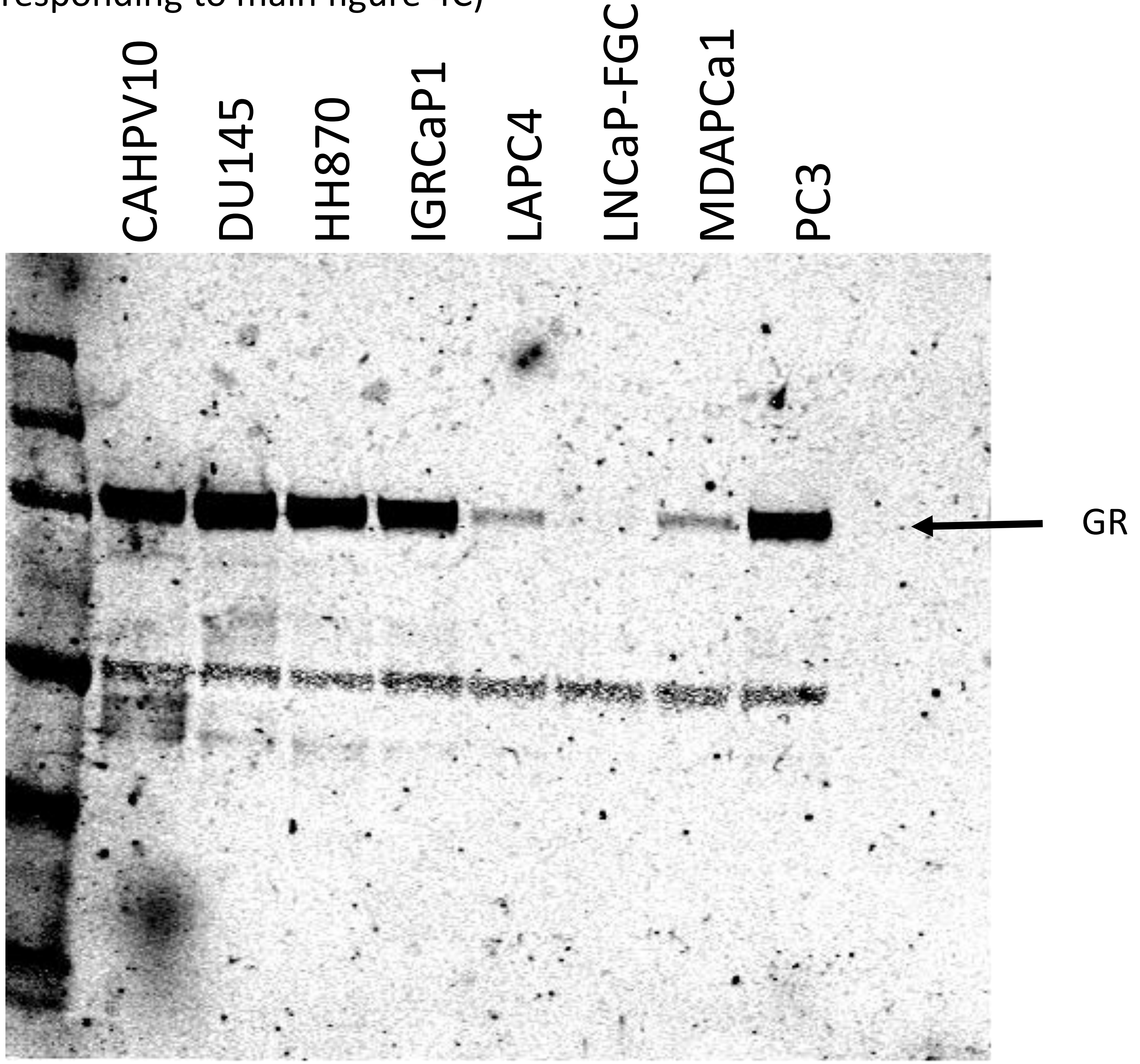

Supplemental Figure S1: β-Tubulin loading control blots for prostate cancer cell lines (corresponding to main figure 4C)

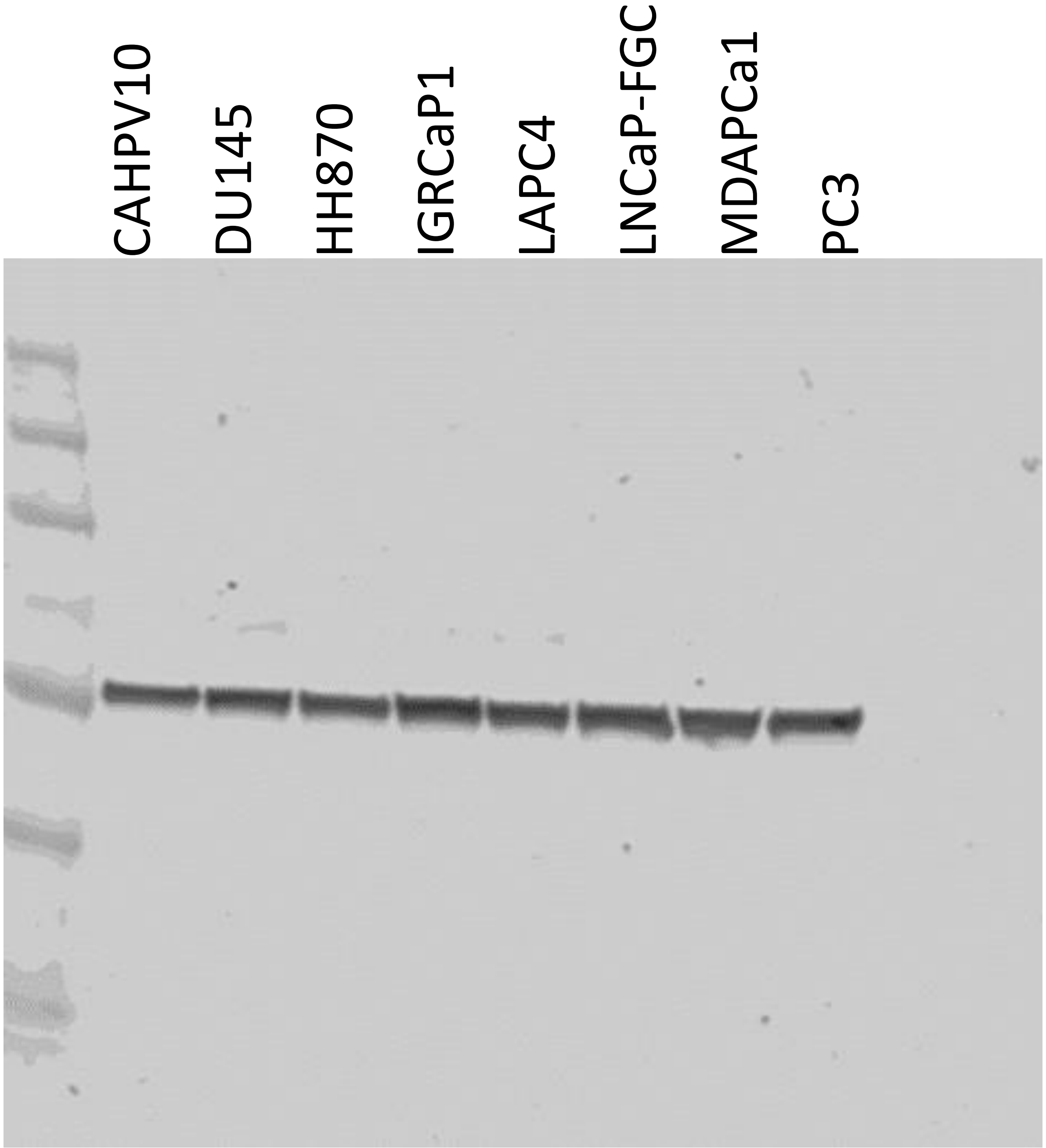

Supplemental Figure S1: GR and β-Tubulin loading control blots for prostate cancer cell lines (corresponding to main figure 4E)

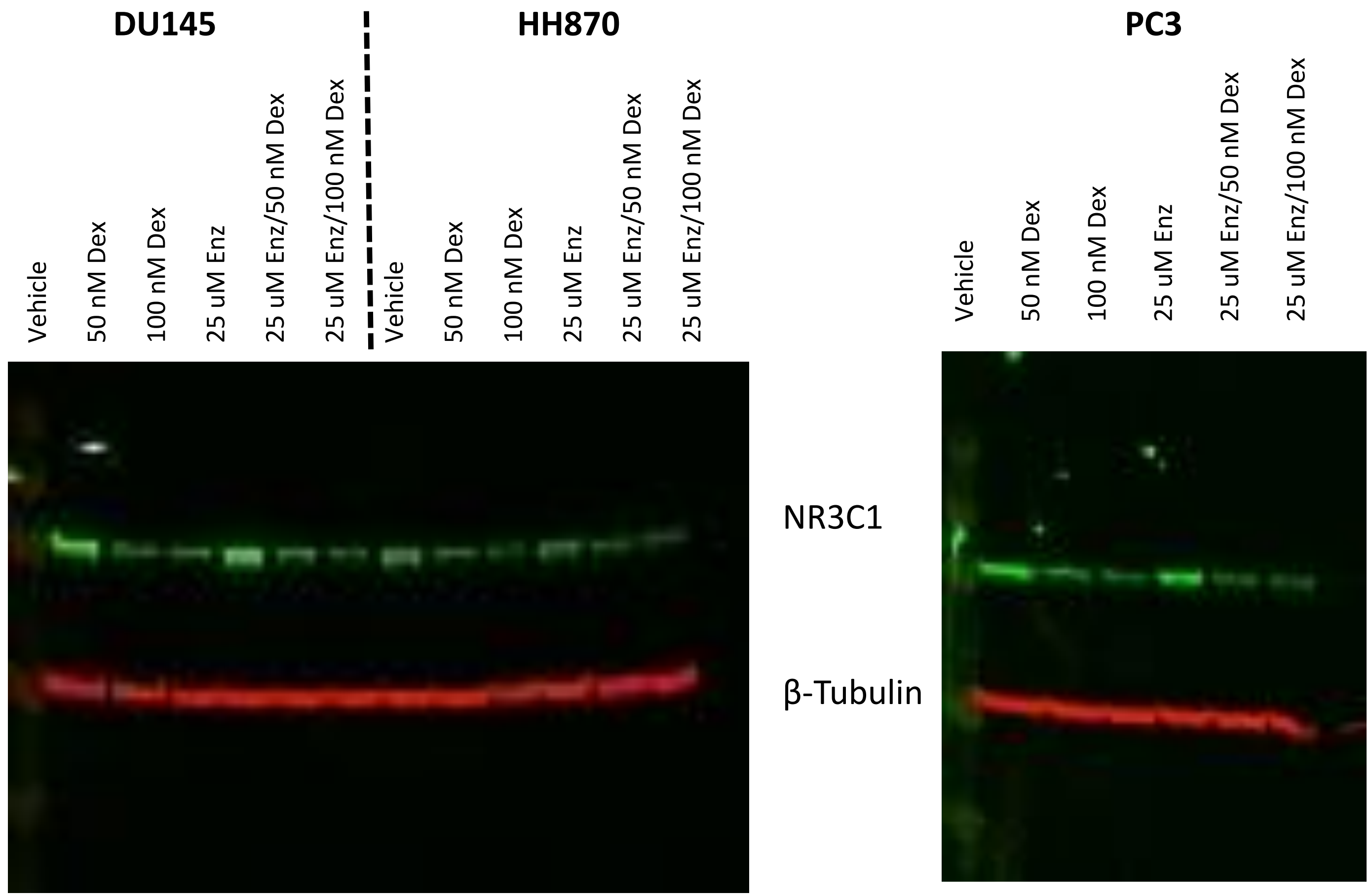

Supplement: Supplementary file 1 — Supplementary Figure S1. [file 41598_2020_78798_MOESM1_ESM.pdf]
